# PREview scores and applicant conduct indicators: distinguishing current professional readiness from historical events

**DOI:** 10.3389/fmed.2026.1884967

**Published:** 2026-08-12

**Authors:** Kenneth D. Royal, Andrea M. Carpentieri, Robert Santos, David W. Stenulson

**Affiliations:** Admissions Analytics and Insights, Association of American Medical Colleges, Washington, DC, United States

**Keywords:** applicant misconduct, medical education, medical education assessment, medical school admissions, PREview, professional readiness, professionalism, situational judgement test

## Abstract

**Introduction:**

Professionalism is a foundational expectation for medical training, yet traditional academic metrics provide limited insight into applicants’ professional readiness. The AAMC PREview® Professional Readiness Exam assesses how applicants evaluate and respond to realistic scenarios requiring effective professional behavior. Prior studies have not examined how PREview scores relate to misconduct indicators in medical school applications. This study compares PREview scores across applicants with and without AMCAS misconduct indicators, focusing on differences between contemporaneous professionalism concerns and historical misconduct after accounting for academic preparation.

**Methods:**

This quasi-experimental observational study analyzed national-level AMCAS applicant data from the 2021–2025 application cycles for U.S. MD-granting medical schools. Analyses were conducted separately for four misconduct indicators: felony conviction, misdemeanor conviction, prior institutional action, and active investigation during the application cycle. Kernel-based propensity score weighting was used to establish academic comparability based on MCAT score, undergraduate GPA, and application year and to estimate average treatment effects on the treated (ATT) for PREview scores.

**Results:**

After adjustment for academic covariates, applicants under active investigation scored an average of 1.48 PREview points lower than academically comparable applicants without an investigation. Associations between PREview scores and historical misconduct indicators were substantially smaller after adjustment, and estimates for felony convictions were imprecise due to rarity.

**Conclusion:**

After accounting for academic preparation, PREview scores were more strongly associated with contemporaneous professionalism concerns than with historical misconduct indicators. This pattern is consistent with the intended interpretation of PREview scores as a forward-looking assessment of applicants’ responses to professionalism-relevant situations in the medical school admissions context.

## Introduction

Professionalism is a foundational expectation for entering medical students, shaping how future physicians interact with patients, colleagues, and learning environments (1–3). Although professionalism lapses among medical trainees are relatively uncommon, their consequences can be substantial for individuals, institutions, and patient safety (4–6). Prior research has shown that non-academic dismissal from medical school, which is often linked to professionalism-related concerns, differs fundamentally from academic forms of attrition. In a recent national analysis, undergraduate GPA and MCAT scores were strong predictors of academic withdrawal and dismissal, whereas the AAMC PREview® Professional Readiness Exam uniquely predicted non-academic dismissal (7). This divergence reinforces the conceptual distinction between academic readiness and professional readiness and suggests that PREview scores are associated with competencies relevant to ongoing professional behavior.

The PREview® exam was intentionally developed to assess professional readiness for medical school through a situation judgment framework grounded in realistic scenarios (8). According to the Association of American Medical Colleges (AAMC), PREview measures two broad skill domains essential for success in medical training: relational skills (e.g., teamwork, communication, empathy, and collaboration) and personal accountability (e.g., ethical responsibility, reliability, resilience, adaptability, and self-improvement). These domains reflect how applicants reason through professional challenges they are likely to encounter in medical school rather than stable personality traits. PREview scores therefore reflect applicants’ understanding of effective and ineffective professional behaviors in context-specific situations, aligning the exam with contemporary competency-based and holistic admissions frameworks.

Despite the emerging evidence base for PREview®, no national studies have examined how PREview scores relate to applicant misconduct indicators collected during the medical school application process. The American Medical College Application Service (AMCAS) includes several applicant- or institution-reported indicators intended to alert admissions committees to potential concerns, including felony convictions, misdemeanor convictions, prior institutional actions, and active investigations arising during the application cycle (9). These categories vary widely in severity, contextual relevance, and institutional reporting practices. Importantly, little is known about whether and how these indicators reflect the professional readiness competencies PREview scores are intended to assess.

Understanding how PREview scores relate to different categories of applicant misconduct has important implications for admissions practice. First, it may clarify the extent to which PREview scores are associated with different categories of applicant behavior observed during the admissions process. Second, it may provide guidance for admissions committees seeking to interpret misconduct indicators fairly and consistently during the application review and admissions process. If PREview scores are more strongly associated with certain forms of misconduct than others, these patterns may help distinguish indicators that reflect ongoing professional readiness concerns from those tied to past or context-dependent events.

The purpose of this study was to evaluate how PREview scores differ among applicants with and without AMCAS misconduct indicators using a large national dataset and a kernel-based propensity score weighting approach. By adjusting for MCAT scores, undergraduate GPA, and application year, this study estimates the extent to which misconduct indicators are associated with PREview scores after accounting for observable differences in academic background.

## Methods

### Study design and participants

This quasi-experimental observational study analyzed national-level AMCAS applicant data from the 2021–2025 admissions cycles for MD-granting medical schools in the United States. The dataset included PREview scores, academic metrics, application year, and applicant- or institution-reported misconduct indicators collected as part of the AMCAS application process. The analytic sample was restricted to applicants with complete data on PREview score, MCAT score, undergraduate GPA, application year, and the misconduct indicator under study.

### Variables

The primary outcome was the PREview score (range, 1–9). PREview® is a situational judgment test (SJT) designed to assess professional readiness, evaluating examinees’ understanding of effective and ineffective professional behaviors across realistic medical-school-relevant scenarios. Consistent with the current AAMC framework, PREview focuses on two overarching skill domains central to professional readiness: relational skills (e.g., communication, teamwork, empathy, and collaboration) and personal accountability (e.g., ethical responsibility, reliability, resilience, adaptability, and reflective self-improvement). Responses are scored relative to consensus effectiveness ratings established by trained medical educators and student affairs professionals, reflecting widely shared standards for professional readiness rather than normative comparison to other applicants. Scores were treated as continuous for all analyses.

Four binary misconduct indicators (yes/no) were examined separately. These included felony conviction, misdemeanor conviction, prior institutional action, and active investigation during the application cycle. Felony, misdemeanor, and institutional action indicators capture historical, administrative or legal events that occurred prior to the application cycle. In contrast, the active investigation indicator reflects ongoing, unresolved concerns arising during the application process itself. Investigations commonly involve issues including false or incomplete reporting, failure to disclose required information, misrepresentation, unprofessional engagement with admissions offices, or other violations of AMCAS policies.

MCAT score and undergraduate GPA were included as covariates to account for differences in academic preparation across applicants. Application year was included to address cohort-level variation. These covariates were specified *a priori* because they are available for all applicants and are plausibly associated with both PREview scores and the presence of misconduct indicators.

### Data analysis

We first provide summary statistics describing the distribution of PREview scores by presenting unweighted mean scores by misconduct category to illustrate baseline differences. The primary analyses employed kernel-based propensity score weighting to estimate average treatment effects on the treated (ATT). Propensity scores were estimated using logistic regression models that included MCAT score, undergraduate GPA, and application year as predictors. Kernel methods were selected because they leverage the full pool of academically comparable control applicants rather than restricting comparisons to a small number of nearest matches (10–12), thereby improving efficiency and stability in settings with rare or unevenly distributed treatment groups (13–15). Gaussian kernels were used, with bandwidths determined by Silverman’s rule of thumb based on the distribution of propensity scores among control applicants. Gaussian kernel weights were calculated using these propensity scores and Silverman’s rule-of-thumb bandwidth. Control weights were normalized to sum to one, and treated applicants were assigned unit weight, consistent with ATT estimation. ATT estimates were obtained using kernel-weighted linear regression models with robust standard errors.

Covariate balance before and after weighting was assessed using standardized mean differences (SMDs) for MCAT score, undergraduate GPA, and application year. Absolute SMD values below 0.10 were used as the criterion for acceptable post-weighting balance.

Alternative matching specifications, including nearest-neighbor matching, were explored during model development to assess the influence of matching strategy on estimated effects. Because the study’s primary inferential framework was pre-specified as kernel-based propensity score weighting, only the kernel-weighted results are presented.

ATT estimates represent differences in PREview scores between applicants with a given misconduct indicator and academically comparable applicants without that indicator. Standardized effect sizes were calculated by dividing ATT estimates by the pooled standard deviation of PREview scores and are reported to facilitate interpretation of effect magnitude rather than as primary inferential metrics (16). Confidence intervals were calculated using robust standard errors from weighted models. All analyses were conducted using Stata 18 (StataCorp, College Station, TX). Statistical significance was evaluated using two-sided tests with an alpha level of 0.05.

### Analytic transparency

To enhance transparency, misconduct indicators, propensity-score covariates, matching procedures, bandwidth-selection methods, and ATT estimation procedures were specified prior to final analyses. Results were generated from documented statistical code and reproducible analytical outputs.

### Research ethics

All applicants included in this study provided consent for their data to be used for research purposes when they completed the AMCAS application, the MCAT® exam, and the PREview® exam, all products owned by the Association of American Medical Colleges (AAMC). This study was deemed exempt by the American Institutes for Research institutional review board (protocol: #2218897-1).

## Results

### Sample and misconduct prevalence

The analytic sample included applicants from the 2021–2025 AMCAS application cycles with complete data on PREview scores, MCAT scores, undergraduate GPA, application year, and misconduct indicators. Treated sample sizes varied substantially across misconduct categories, ranging from 12 applicants with reported felony convictions, 622 applicants with misdemeanor convictions, 4,636 applicants reporting prior institutional actions, and 124 applicants identified as being under active investigation during the application cycle. Unweighted PREview mean scores by misconduct indicator are presented in Table 1.

**Table 1:** Unweighted PREview scores by misconduct indicator.

Misconduct indicator	*N*	PREview mean (SD)
Felony		
Conviction	12	4.58 (2.02)
No conviction	77,435	5.41 (1.96)
Misdemeanor		
Conviction	622	5.06 (2.01)
No conviction	76,825	5.41 (1.96)
Prior institutional action		
Prior institutional action	4,636	5.22 (1.99)
No action	72,811	5.42 (1.96)
Active investigation		
Active investigation	124	4.03 (2.15)
No investigation	77,323	5.41 (1.96)

### Covariate balance

Prior to matching, standardized mean differences (SMDs) between treated and control applicants indicated substantial covariate imbalance across all misconduct categories, particularly for MCAT scores and undergraduate GPA (e.g., SMDs ≥ |0.40| for misdemeanor convictions and ≥|1.10| for applicants under active investigation). Following kernel-based propensity score matching, covariate balance improved markedly across all analyses. Post-matching SMDs were substantially reduced across all analyses. Most post-matching SMDs fell below the commonly cited 0.10 threshold, while the remaining values only slightly exceeded this benchmark (Table 2), indicating markedly improved covariate balance. These results indicate strong comparability between treated applicants and kernel-weighted control applicants on observed academic characteristics.

**Table 2:** Standardized mean differences (SMDs) before and after kernel matching.

Misconduct indicator	Covariate	SMD before	SMD after
Felony conviction	MCAT score	−0.94	−0.01
	Undergraduate GPA	−0.59	0.13
	Application year	−0.36	0.00
Misdemeanor conviction	MCAT score	−0.08	0.02
	Undergraduate GPA	−0.41	0.01
	Application year	−0.25	−0.01
Prior institutional action	MCAT score	−0.17	−0.02
	Undergraduate GPA	−0.67	−0.01
	Application year	0.03	−0.04
Active investigation	MCAT score	−0.76	−0.13
	Undergraduate GPA	−1.10	0.01
	Application year	0.03	−0.03

Standardized mean differences (SMDs) compare treated and control applicants on matching covariates. Absolute SMD values below 0.10 are commonly interpreted as indicating acceptable balance. Kernel-based propensity score matching substantially reduced imbalance across academic and cohort characteristics for all misconduct indicators.

### PREview scores by misconduct Indicator

Unmatched comparisons showed lower mean PREview scores among applicants with misconduct indicators relative to other applicants. After kernel-based matching, control group PREview means reflected weighted counterfactual estimates for academically similar applicants, enabling estimation of average treatment effects on the treated (ATT).

### Kernel-based ATT estimates

Kernel-based propensity score matching revealed heterogeneity in PREview score differences across misconduct indicators (Table 3). On average, the mean PREview scores of applicants under active investigation during the application cycle were 1.48 points lower than those of matched applicants without an investigation (95% CI, −1.86 to −1.11), corresponding to a standardized effect size of −0.74.

**Table 3:** PREview score differences: Unmatched vs. kernel-matched.

Misconduct indicator	Treated *n*	ATT (PREview points)	95% confidence interval	Standardized effect size
Felony conviction	12	−1.10	[−2.19, −0.00]^†^	−0.55
Misdemeanor conviction	622	−0.48	[−0.64, −0.32]	−0.24
Prior institutional action	4,636	−0.34	[−0.40, −0.28]	−0.17
Active investigation	124	−1.48	[−1.86, −1.11]	−0.74

Estimates represent Average Treatment Effects on the Treated (ATTs) derived from Gaussian kernel propensity-score matching using propensity scores estimated from MCAT score, undergraduate GPA, and application year. ATTs were estimated using kernel-derived weights and weighted linear regression with robust standard errors. Standardized effect sizes were calculated by dividing ATT estimates by the pooled PREview score standard deviation.

^†^Interpret with caution because of the very small number of applicants reporting a felony conviction (*n* = 12).

Mean PREview scores among applicants reporting prior institutional actions were 0.34 points lower than those of matched control applicants (95% CI, −0.40 to −0.28), corresponding to a modest standardized effect size (−0.17). Mean PREview scores among applicants reporting misdemeanor convictions were 0.48 points lower than those of matched control applicants (95% CI, −0.64 to −0.32), corresponding to a small standardized effect size (−0.24). Estimates for felony convictions were imprecise due to the very small treated sample size (*n* = 12) and the resulting wide confidence interval, and should be interpreted cautiously.

## Discussion

Because professionalism is a foundational expectation for medical training, understanding how PREview scores relate to different forms of applicant misconduct has direct implications for admissions decision-making. This study demonstrates a clear and meaningful distinction between contemporaneous professionalism concerns and historical misconduct indicators after adjustment for academic background. This distinction has important implications for how PREview scores should be interpreted and used in admissions.

### Current versus historical misconduct

The most pronounced and robust PREview score differences were observed for the group of applicants under active investigation, whose mean PREview scores were 1.48 points lower than academically comparable applicants without an investigation. This association was large in magnitude, precisely estimated, and consistent across analytic approaches. In contrast, associations between PREview scores and historical misconduct indicators were generally smaller after adjustment, particularly for prior institutional actions and misdemeanor convictions. Collectively, these results indicate that PREview scores are more strongly associated with contemporaneous professionalism concerns than with past administrative or legal events alone. This pattern is consistent with the exam’s design as a forward-looking measure of how applicants evaluate and respond to professional challenges they are likely to encounter in medical training.

### Interpreting PREview scores in admissions-relevant comparisons

Kernel-based matching was selected as the primary analytic approach because it leverages the full pool of academically comparable applicants rather than restricting comparisons to a limited number of nearest matches. This approach is particularly useful when treatment groups are rare or unevenly distributed and more closely reflects the broader comparison framework typically used in admissions decision-making.

### Interpreting PREview scores in light of behavioral theory

The observed distinction between current and historical misconduct aligns with well-established theoretical perspectives emphasizing the developmental and contextual nature of behavior. Developmental (17–19) and personality (20–23) research consistently demonstrates that behavior changes over time and that early actions may reflect situational or developmental contexts rather than stable dispositions. Similarly, professional identity formation literature highlights that professional values and behaviors consolidate over time, often emerging later than pre-professional experiences (24).

From a person–environment perspective, behavior reflects ongoing interactions with current contexts and expectations (25). Active investigations, by definition, capture unresolved concerns within the applicant’s present environment, making them theoretically more informative indicators of current professional functioning. Historical misconduct indicators, in contrast, often reflect past environments, administrative processes, or circumstances that may not translate directly to present readiness for medical training. The stronger association of PREview scores with contemporaneous professionalism concerns, relative to historical misconduct indicators, is consistent with these theoretical perspectives.

### Validity considerations

Viewed through a unified construct-validity perspective (26, 27), this pattern of results provides validity evidence consistent with the intended interpretation of PREview scores. PREview scores were more strongly associated with contemporaneous professionalism concerns than with historical misconduct indicators, a pattern that is theoretically consistent with the exam’s focus on professional judgment and decision-making in present or near-future contexts. Historical misconduct indicators may reflect prior circumstances, developmental stages, institutional processes, or contextual factors that are less directly connected to applicants’ current approaches to professional situations. These findings align with the AAMC’s intended interpretation and use of PREview scores in admissions decision-making rather than treating scores as proxies for generalized misconduct history.

### Alignment with prior outcome research

Prior national research shows that PREview scores are strongly associated with non-academic dismissal from medical school, whereas MCAT scores and undergraduate GPA are more strongly associated with academic dismissal (7). Because non-academic dismissal is typically driven by professionalism-related concerns (e.g., reliability, ethical judgment, interpersonal behavior, and adherence to professional standards), the present findings offer upstream insight into patterns that may be later observed in training. PREview scores were more strongly associated with indicators of current professional functioning, such as active investigation status, than with historical misconduct alone. This coherence across selection-stage indicators and training-stage outcomes strengthens confidence in the interpretive meaning of PREview scores.

### Relationship to prior SJT research

Research on situational judgment tests (SJTs) has generally reported modest and context-dependent associations with professionalism-related outcomes. For example, a single-institution study examining PREview scores reported no association with post-interview committee evaluations (28), whereas a study conducted in the United Kingdom found only modest associations between performance on the University Clinical Aptitude Test (UCAT) situational judgment test and subsequent disciplinary outcomes among medical students (29). Recent meta-analytic evidence further suggests that SJT validity is influenced by the degree of construct congruence between the assessment and the outcome being evaluated, with stronger associations observed when outcome measures more closely reflect the personal and professional attributes targeted by the assessment (30). These findings suggest that relationships between SJT performance and downstream outcomes may vary according to the outcome examined, institutional context, stage of training, and assessment used. The present study contributes to this broader literature by examining applicant misconduct indicators within a national admissions sample and by distinguishing between contemporaneous professionalism concerns and historical misconduct indicators.

### Implications for applicant review and admissions

For admissions committees and policymakers, these findings highlight the importance of interpreting misconduct information with careful attention to timing and context. PREview scores offer useful insight into how applicants evaluate and respond to professionalism-relevant situations while showing more modest associations with historical misconduct indicators once academic differences are accounted for. In contrast, groups of applicants with ongoing professionalism concerns, such as active investigations during the application cycle, demonstrate substantially lower mean PREview scores, on average. When used as intended, PREview scores provide additional information regarding applicants’ responses to professionalism-relevant situations, complementing academic metrics that capture academic readiness and predict academic performance.

### Limitations

Several limitations should be considered when interpreting these findings. First, this study relied on applicant-level AMCAS data and did not observe downstream admissions outcomes, such as interview offers, acceptances, or matriculation. Accordingly, conclusions are limited to how PREview scores differ across applicant misconduct categories, not how those scores ultimately influence admissions decisions. Second, misconduct indicators are self- or institution-reported and may vary across institutions in consistency, interpretation, and reporting practices. Historical categories such as felony convictions, misdemeanor convictions, and prior institutional actions encompass a wide range of behaviors, severities, and contexts that cannot be distinguished using binary indicators alone. Similarly, although active investigation status reflects contemporaneous concerns, the specific circumstances underlying these investigations were not directly observed. More broadly, the binary misconduct indicators likely aggregate heterogeneous behaviors that vary considerably in severity, context, and relevance to professional functioning. As a result, estimated effects should be interpreted as average effects across heterogeneous categories rather than associations with specific forms of misconduct.

Third, analyses were restricted to applicants who completed the PREview examination. Applicants who completed PREview may differ systematically from those who did not, and findings may therefore not generalize to the broader AMCAS applicant population. Fourth, although kernel-based propensity score weighting adjusted for key measures of academic preparation and cohort effects, unmeasured confounds remain possible. Factors such as prior life experiences, socio-demographic characteristics, psychosocial stressors, or institutional climate may influence both misconduct reporting and PREview score performance and were not available for analysis. Fifth, misconduct indicators were rare relative to the overall applicant population, particularly for felony convictions, resulting in limited precision for some estimates despite the use of robust matching methods. Findings for these rare categories should therefore be interpreted cautiously, particularly for felony convictions, where only 12 applicants met inclusion criteria. Finally, this study is observational in nature and does not support causal inference. The results describe statistical associations between PREview scores and misconduct indicators after balancing observable covariates and should not be interpreted as demonstrating that PREview directly measures professional readiness or causes differences in applicant behavior.

## Conclusion

After accounting for academic preparation, PREview scores showed a strong association with contemporaneous professionalism concerns and substantially weaker associations with historical misconduct indicators. Viewed together, this pattern provides validity evidence consistent with the intended interpretation of PREview scores and supports a forward-looking, construct-aligned interpretation of PREview scores during application review.

## Data Availability

The datasets presented in this article are not readily available because they are derived from restricted AAMC data sources that cannot be publicly shared. Requests for access to de-identified data may be considered on a case-by-case basis, subject to approval by the AAMC. Requests to access the datasets should be directed to Dr. Kenneth Royal (kroyal@aamc.org).
